# RACE, NEIGHBORHOOD DYNAMICS, AND MORTALITY PATTERNS IN OLDER PUERTO RICANS

**DOI:** 10.1093/geroni/igae098.0313

**Published:** 2024-12-31

**Authors:** Catherine García, Maria Aranda, Michael Crowe

**Affiliations:** Syracuse University, Syracuse, New York, United States; University of Southern California, Los Angeles, California, United States; University of Alabama at Birmingham, Birmingham, Alabama, United States

## Abstract

This study explores the relationships between race, neighborhoods, and mortality among older Puerto Ricans, drawing upon theoretical frameworks in sociology, gerontology, and public health. We combined data from the longitudinal Puerto Rican Elderly Health Conditions Project (PREHCO; 2002-2021) and the Puerto Rico Contextual Data Resource (PR-CDR) to examine how the relationships between racial identity, neighborhood-level racial density, and neighborhood-level socioeconomic status are related to the probability of experiencing all-cause mortality among island-dwelling Puerto Ricans aged 60 and older (n=4,243). Findings show that young-old Black Puerto Ricans residing in high socioeconomic neighborhoods have a lower risk of mortality relative to their same aged Black counterparts residing in low socioeconomic neighborhoods, and White Puerto Ricans who reside in either high or low socioeconomic neighborhoods. However, this mortality advantage diminishes when they reach the age of 75 years and over. We hypothesize that in Puerto Rico, Black communities with access to economic capital are especially beneficial for its residents as they are in spaces where they feel welcomed and are surrounded by amenities that are essential for healthy aging or survival into old age. As Puerto Ricans in these communities age, this advantage may diminish, due to a ceiling effect, meaning that for older Puerto Ricans who survive to age 60 in these communities, there are fewer advantages to accumulate. Our research provides a foundation for informed policy decisions and targeted interventions to promote aging in place in Puerto Rico and offers a framework for understanding similar dynamics in diverse Latin American contexts.

